# Genetic characterization and meta-analysis of the population of the Northern Black Sea region in the 1st millennium CE based on ancient DNA data

**DOI:** 10.18699/vjgb-26-66

**Published:** 2026-07

**Authors:** E.D. Aituganova, A.S. Kon’kov, T.V. Andreeva, E.V. Rozhdestvenskikh, A.D. Manakhov, E.I. Rogaev

**Affiliations:** Sirius University of Science and Technology, Sirius Federal Territory, Krasnodar region, Russia; Sirius University of Science and Technology, Sirius Federal Territory, Krasnodar region, Russia; Sirius University of Science and Technology, Sirius Federal Territory, Krasnodar region, Russia Vavilov Institute of General Genetics of the Russian Academy of Sciences, Moscow, Russia Centre of Genetics and Genetic Technologies, Department of Genetics, Faculty of Biology, Lomonosov Moscow State University, Moscow, Russia; Sirius University of Science and Technology, Sirius Federal Territory, Krasnodar region, Russia; Sirius University of Science and Technology, Sirius Federal Territory, Krasnodar region, Russia Vavilov Institute of General Genetics of the Russian Academy of Sciences, Moscow, Russia; Sirius University of Science and Technology, Sirius Federal Territory, Krasnodar region, Russia Department of Psychiatry, UMass Chan Medical School, Shrewsburry, MA, USA

**Keywords:** archaeogenetics, ancient DNA, genome, Northern Black Sea region, Sarmatians, Chernyakhov culture, Alans, Bulgars, Saltovo-Mayaki culture, археогенетика, древняя ДНК, геном, Северное Причерноморье, сарматы, черняховская культура, аланы, булгары, салтово-маяцкая культура

## Abstract

Over the past two decades, the introduction of whole-genome sequencing analysis of ancient DNA has led to a breakthrough in archaeogenetic research, significantly expanding our understanding of human genetic history. In this context the Northern Black Sea region during first millennium CE (1–1,000 CE) is of particular relevance, as it remained a hub of intense cultural exchange and migration. Despite its historical importance, ancient genomic data from this period remains scarce, and a comprehensive synthesis of existing findings is lacking. This study presents a systematic review and meta-analysis of published whole-genome sequencing data from 48 ancient samples associated with key archaeological cultures of the region: Late Scythian, Sarmatian, Alan, Bulgar, Saltovo-Mayaki and Chernyakhov. Through the systematization of data, we trace genetic continuity at Late Scythian and Alanian sites relative to preceding populations. Episodes of large-scale migration and population replacement have been documented, most clearly evident in the Sarmatian expansion of the 1st–4th centuries CE, with genetic traces extending from the Urals to the Carpathians. Based on limited evidence, genetic continuity has been identified between representatives of the Chernyakhov culture and early Slavic groups. Through our meta-analysis, we further detect intercultural connections between the Alans, Bulgars, and bearers of the Saltovo-Mayaki culture, whose genetic structure reveals the influence of Caucasian and East Eurasian components. Collectively, these findings underscore the complex genetic landscape of the region, shaped by successive migration waves and multifaceted intercultural contacts. We conclude by outlining key unresolved questions and future directions for archaeogenetic research in the Northern Black Sea region during the first millennium CE.

## Abstract (ru)

За последние два десятилетия внедрение полногеномного анализа древней ДНК привело к прорыву в археогенетических исследованиях, существенно расширив наше понимание генетической истории человечества. На этом фоне особую актуальность приобретает изучение Северного Причерноморья I тысячелетия нашей эры (1–1000 гг.), региона, который в течение всего периода оставался зоной интенсивного культурного обмена и миграций. Несмотря на его историческую значимость, объем доступных данных древней ДНК по этому периоду пока остается ограниченным. В настоящей работе систематизированы и проанализированы опубликованные результаты полногеномного секвенирования 48 древних образцов, относящихся к различным археологическим культурам региона: позднескифской, сарматской, аланской, булгарской, салтово-маяцкой и черняховской. Систематизация данных и применение методов популяционно-генетического анализа позволили проследить генетическую преемственность на памятниках позднескифской, а также отдельно – аланской культуры по отношению к предшествующему населению. Удалось обнаружить межкультурные связи алан, булгар и носителей салтово-маяцкой культуры, в генетической структуре которых отмечается влияние кавказского и восточноевразийского компонентов. С другой стороны, зафиксированы эпизоды масштабных миграций и полной смены населения, наиболее ярко выраженные в сарматской экспансии I–IV вв. н. э., следы которой генетически прослеживаются от Урала до Карпат. По единичным образцам выявлена генетическая преемственность между представителями черняховской культуры и ранними славянскими группами. Полученные результаты демонстрируют мозаичность генетического ландшафта региона, формировавшегося под воздействием многоволновых миграций и межкультурных контактов. Статья освещает современное состояние исследований, показывает основные достижения и намечает перспективы дальнейшего изучения в области археогенетики Северного Причерноморья в I тысячелетии н. э.

## Introduction

For centuries, the Northern Black Sea region, as part of the Pontic-Caspian steppe, has been characterised by high population mobility, which has shaped its complex demographic and ethno-cultural history.

During this period, the population of the Northern Black Sea coast consisted of Hellenised groups who, in the 7th–5th centuries BC, founded numerous poleis: Olbia, Chersonesos, Panticapaeum, Phanagoria and others (Koshelenko et al., 1984). In the first centuries CE, these centers maintained a stable antique cultural zone, sustaining close contacts both with the metropolitan centers and with the local population – Scythians, Taurians, Maeotians, Sindi, and Getae (Alekseeva, 1991). Throughout the first millennium they fell within the sphere of influence of Rome, Byzantium, and later the Khazar Khaganate (Pletneva, 2003; Afanasyev et al., 2015a).

At the same time, the region’s steppe lands became a corridor for successive waves of migration by nomadic tribal confederations. By the beginning of the first millennium CE, groups of the Sarmatian culture began to succeed the European Scythians, establishing dominance in the region and extending their influence from the Don to the Danube (Mordvintseva, 2013; Koryakova, 2018; Kovács, 2023). Later, in the 1st–3rd centuries, the Alans came to dominate the Azov region and the Ciscaucasia (Perеvalov, 2014). From the late 2nd to the early 3rd century, Germanic tribes expanded into the region from northwestern Europe. These groups left a distinct imprint on the cultural and political structure of the Northern Black Sea region’s population, contributing to the formation of the Chernyakhov culture (3rd–5th centuries) (Kazanski, 2011; Matveev, 2017; Onishchuk, 2018).

A major turning point in the demographic history of the region came with the Hunnic invasion in the 4th–5th centuries, which marked the beginning of the Migration Period and was accompanied by large-scale population movements (Shushunova, Yartsev, 2024). In the 5th–7th centuries, the Huns were succeeded by nomadic confederations of the Onogurs, Kutrigurs, Savirs, and later the Avars (Bubenok, 2014). In the mid-7th century, Great Bulgaria emerged in the region, soon to be replaced by the Khazar Khaganate (Pletneva, 2003; Afanasyev et al., 2015a; Kazanski, 2020). From the late 7th to the 8th centuries, the Khazar Khaganate became the dominant force in the region, controlling the steppes from the Don to the Caucasus, as well as the eastern Crimea and the Taman Peninsula. Within these territories, under Khazar control, the Saltovo-Mayaki culture also developed (Pletneva, 2003; Berezina et al., 2012; Reshetova, 2012; Afanasyev et al., 2015a). Concurrently, in the more northern forest-steppe areas of the region, the settlement of Slavic tribes began (Sedov, 1979).

The complexity and multi-layered nature of the historical and cultural processes in the Northern Black Sea region have long attracted the attention of researchers. In recent years, the reconstruction of the region’s population history has increasingly been based on the analysis of ancient DNA using a range of population genetics methods. In autosomal analysis, the principal component method is used to visualise the structure of genetic diversity (Patterson et al., 2006; McVean, 2009), while the ADMIXTURE method is employed to identify likely ancestral components (Alexander et al., 2009). Population origin models based on f-statistic methods are used, which measure the covariance of allele frequencies, thereby allowing the estimation of genetic distances, the identification of mixed ancestry, and the direction of gene flow (Patterson et al., 2012).

Based on these principles, the qpAdm and qpWave tools have been developed, which are widely used to test demographic hypotheses and quantify admixture proportions (Haak et al., 2015; Harney et al., 2021). IBD analysis identifies regions of the genome inherited from common ancestors, enabling the assessment of genealogical relationships between individuals (Ringbauer et al., 2024). Analysis of mitochondrial DNA (mtDNA) and Y-chromosome haplogroups allows for a detailed reconstruction and investigation of maternal and paternal lineages.

Despite the rapid growth in archaeogenetic research, data from the Northern Black Sea region remain fragmentary and unevenly represented both chronologically and culturally. Consequently, the systematization, comparison, and meta-analysis of published data become particularly critical. This approach allows for the identification of patterns in genetic dynamics, assessment of the robustness of proposed models of origin and migration, and outlining the limitations of the current state of research as well as future perspectives.

**Fig. 1.**
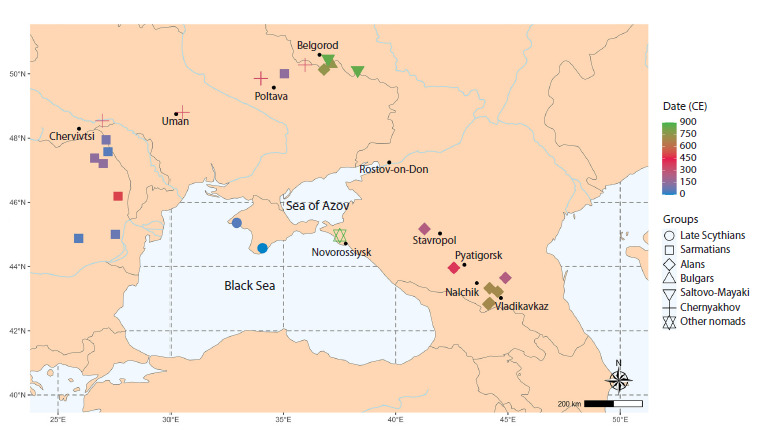
Geographic location of the archaeological sites from which 48 ancient individuals used in this study Samples from archaeological sites are indicated by symbols corresponding to their respective archaeological culture. The color scale indicates sample dating, based on archaeological chronology or radiocarbon analysis (150-year intervals, spanning 0 to 900 CE). Scale bar: 200 km.

In this study, we conducted a systematic review and metaanalysis of published whole-genome ancient DNA data from the Northern Black Sea region dating to the 1st millennium CE (Fig. 1).

## Materials and methods

Sources were selected based on their peer-reviewed status and the availability of reliable data regarding the archaeological context, geographic provenance, and dating of the samples. We used archaeogenetic descriptions and genomic data from articles and/or AADR v62.0 database (Mallick et al., 2024), followed by downloading genomes from the ENA resource (ENA Browser, 2025).

Principal component analysis (PCA) was performed using the smartpca function of the EIGENSOFT package (Patterson et al., 2006). The genotypes of the studied samples were projected onto the genetic variation space of more than 1,300 individuals from 80 present-day European and Caucasian populations from the Human Origin panel, comprising approximately 600 thousand SNPs (Lazaridis et al., 2016). To estimate the proportions of ancestral origins in the genome structure, we used the ADMIXTURE v1.3 software package with the same Human Origin panel (Alexander et al., 2009).

All transitional variants that could represent postmortem modifications, as well as loci with a high proportion of missing data (--geno 0.999), were removed from the dataset. To ensure marker independence, linked SNPs were excluded from the analysis using the PLINK command --indep-pairwise 50/10/0.1. To estimate the optimal number of clusters K, 12 independent calculations were performed in a crossvalidation mode, and the final value of K was taken based on the minimum cross-validation error. Prior to analysis, samples with fewer than 10 thousand markers from the Human Origin panel were excluded.

## Results

In total, we analyzed 48 published ancient genomes from this region, which were grouped based on origin, geography, time period, and archaeological culture (Fig. 1 and see the Table).

**Table 1.**
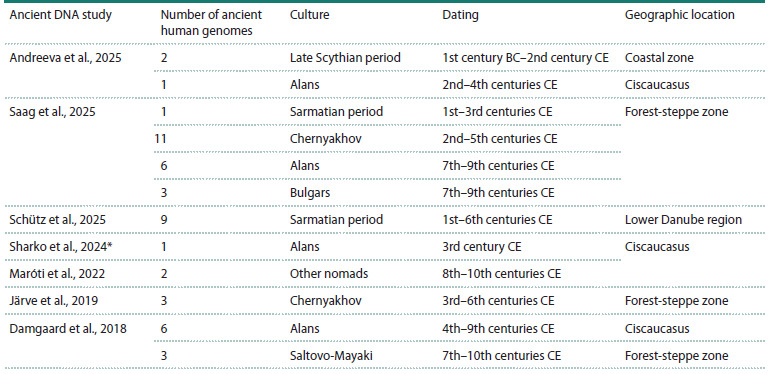
List of published whole-genome data for ancient individuals from the Northern Black Sea region * No meta-analysis was conducted for the individual described in this article due to the poor quality of the genomic data.

Systematic review of published data

This section presents a systematic review of published archaeogenetic studies, grouped by archaeological culture

Late Scythian population. Whole-genome data on the Late Scythian population are presented in only one published study (Andreeva et al., 2025), which includes two individuals from the 1st century BC to the 2nd century CE from the most representative sites of the Late Scythian period – Neapolis Scythian and the Belyaus settlement. Archaeological data from Belyaus indicate a transformation of the site from a Greek settlement into a center of Late Scythian culture, likely accompanied by a change in the population (Dashevskaya, 2014).

The results of principal component analysis (PCA) reveal differences in the genetic profiles of individuals from these sites. One of them (AS10) is similar to other Scythian samples from the Crimea, dating from both the Late Scythian period (1st century BC–1st century CE) and the earlier period (6th– 3rd centuries BC), indicating a probable continuity of the local population (Andreeva et al., 2025). According to the results of qpAdm modelling, his genetic profile, unlike that of other Scythian groups, includes a high proportion (around 40 %) of the Anatolian component, suggesting a connection between the Crimean Scythians and the ancient population of Anatolia. The second individual (AS1) is clustered in the PCA with the classical Scythians of the Northern Black Sea region from the 4th–3rd centuries BC, and his genetic profile contains a significant proportion of an ancient Siberian component (Andreeva et al., 2025).

Collectively, these archaeogenetic data indicate pronounced genetic heterogeneity of the Late Scythian population of the Northern Black Sea region (Andreeva et al., 2025). However, the limited sample size and the isolated nature of research into this period do not allow for generalisations at the level of archaeological culture. It is noteworthy that this study represents the first attempt to predict phenotypic traits for the Late Scythian population of this period using markers from the HIrisPlex-S system (Andreeva et al., 2025). The results indicate a probable brown (chestnut) hair color, intermediate skin tone and brown eyes (Andreeva et al., 2025). Although genomic coverage allowed phenotypic characteristics to be determined for only one individual, these results provide a basis for a subsequent description of the physical appearance of the entire group.

Sarmatian period. In the forest-steppe zone of the Northern Donets, a single individual from the Sarmatian period (1st– 3rd centuries CE) was excavated from the “Lyubovka” burial ground. The burial was located within the cultural layer of an earlier Scythian settlement, but was attributed to the Sarmatian period on the basis of the artefacts found and dated to the 1st–3rd centuries. According to the results of PCA and ADMIXTURE, this individual proved to be similar to the population of the Caucasus 7th–9th centuries CE, as were the later groups of Alans and Steppe Bulgars of the Saltovo- Mayaki culture discussed in this article (Saag et al., 2025).

Interesting findings have emerged from the analysis of genomes from individuals dating to the Sarmatian period (1st–4th centuries CE) from the Lower Danube region (Romania) (Schütz et al., 2025). Analysis using f4, PCA and ADMIXTURE revealed their marked genetic similarity to the steppe Sarmatians of the Urals and Central Asia, and qpAdm modeling results also indicated a high proportion of the steppe component. It is noteworthy that the Sarmatians from the territory of Romania and the Carpathian Basin demonstrate an extremely low proportion of common ancestry with the preceding Scythian-era population from the same territory.

Analysis of chromosomal segments of shared origin (IBD) confirms this picture: extensive shared chromosomal regions were identified among carriers of the Sarmatian culture in the European and Asian parts of the Black Sea–Caspian steppe. In particular, shared IBD segments of up to 64 cM in length were identified between individuals from the Carpathian Basin (POG-10, male, 1st century CE) and from the Rostov Oblast (DA139, female, 8th century BCE–1st century CE). Moreover, individual DA139 shared 88 cM of IBD with a Sarmatian from the Orenburg Oblast (chy001, female, 1st–3rd centuries CE) (Schütz et al., 2025). These data indicate recent common ancestry among these individuals and active migration processes between the eastern and western regions of the Sarmatian world in the early centuries of the 1st millennium CE.

Alans. The Alan population of the Northern Black Sea region studied to date is represented by two main groups, differing both geographically and culturally-archaeologically (see the Table). The first group includes eight Alans from the Ciscaucasus – the population of the mountainous and foothill areas of the North Caucasus. The second group is represented by six Alans from the forest-steppe zone, who inhabited the interfluve of the Seversky Donets and Don rivers and are archaeologically attributed to the Saltovo-Mayaki culture (7th–9th centuries CE).

The earliest archaeogenetic data were obtained from the Ciscaucasia (North Ossetia – Alania) using a sample of six individuals (Damgaard et al., 2018). The authors discussed the origins of the Alans and their presumed Iranian-speaking affiliation. The results revealed no significant Sarmatian contribution to the gene pool of the 5th-century Alans of the Ciscaucasus. Statistical analysis indicated that the North Caucasian Alans form a clade with the Iron Age population from Armenia, demonstrating significant genetic differences between Sarmatians and Alans. The authors suggest that the Alans likely inherited the Iranian language from the steppe Sarmatians, but by the 5th–6th centuries CE, the genetic structure of their population was already virtually indistinguishable from the local Caucasian population.

In the study where the genome of one Alan individual from the kurgan burial ground “Bratskie I Kurgans” (the Chechen Republic) was sequenced (Sharko et al., 2024), different conclusions were drawn. According to PCA results, this individual shows genetic similarity Alans from the Ciscaucasus studied in an earlier work (Damgaard et al., 2018). ADMIXTURE analysis and f3 and f4 statistics results showed that his genome is structurally close to populations of the Koban culture; however, unlike the highland Caucasian groups of the Bronze Age (Maykop and Kura-Araxes cultures), it exhibits the presence of a steppe component. In this study, signs of contamination were identified in some samples, therefore the obtained data require caution in interpretation and subsequent confirmation using other samples and datasets.

The genetic profile of one Alan individual from the site “Solnechnodolsk- 4” (Stavropol region) (radiocarbon age 132–329 cal CE) includes steppe components of Bronze Age populations; according to PCA and ADMIXTURE results, it was found to be similar to the profiles of representatives of the Sarmatian and Koban populations from the same site (Andreeva et al., 2025), which may also indicate population continuity in this region during the transition of archaeological cultures.

Pronounced genetic heterogeneity was documented in the study of a sample of six Alans, bearers of the Saltovo-Mayaki culture, from the forest-steppe zone of the Northern Donets region at the archaeological complex “Verkhniy Saltov” (7th– 9th centuries CE) (Saag et al., 2025). Four individuals from this site demonstrate maximum genetic similarity to populations of the North Caucasus, consistent with the studies described above. Two other individuals exhibit the greatest genetic similarity to populations of Central Asia (Saag et al., 2025).

Taken together, the available data point to a complex and heterogeneous genetic structure within the Alanian population, comprising steppe, North Caucasian and Central Asian genetic components. The inconsistency of some of the results highlights the need to expand the sample sizes and conduct further research on Alanian groups.

Bulgars. Currently, the Bulgar population of the Black Sea region in the 1st millennium CE is represented in genetic studies by only three individuals from the site “Bochkov” in the Seversky Donets basin (Saag et al., 2025). In the genetic profile of these Bulgars, admixture from populations of the Caucasus and Central Asia was identified. The high degree of genetic similarity between the Alans and Bulgars, who are bearers of the same Saltovo-Mayaki culture but exhibit differences in burial rites (catacomb and pit burials, respectively), leads to the conclusion that both groups may have originated from a single genetically homogeneous group. This conclusion aligns with archaeological interpretations, according to which both catacomb and pit burials of the Saltovo-Mayaki culture in the forest-steppe zone are associated with migrations of Alans from the Caucasus (Berezina et al., 2012; Afanasyev et al., 2015b; Korobov, 2019; Malashev, Krivosheev, 2024)

According to qpAdm proximal modeling results, the Sarmatian, Alan, and Bulgar groups from the forest-steppe zone that demonstrate genetic connections to the Caucasus can be modeled by the contribution of a preceding group of elite Scythian nomads from the Seversky Donets basin (Saag et al., 2025).

Three samples of representatives of the Saltovo-Mayaki culture, associated with one of the population groups of the Khazar Khaganate, were studied in an earlier work (Damgaard et al., 2018). These individuals, although heterogeneous, also cluster in the principal component space with Caucasian Alans and other samples from the North Caucasus, and according to a later study, they were found to be related to the forest-steppe zone Alans based on f4 test results (Saag et al., 2025)

Chernyakhov culture. Archaeogenetic studies of the Chernyakhov culture population cover samples from the foreststeppe zone of the region, dating to the 3rd–5th centuries CE. In 2018, the genomes of three Chernyakhov individuals were presented (Järve et al., 2019), and later the sample was supplemented with an additional 11 specimens (Saag et al., 2025).

Results of principal component analysis and qpAdm indicate the identification of at least three genetic groups among representatives of the Chernyakhov culture. One of them includes individuals with a predominance of Eastern and Central European components, the second consists of bearers of a genetic profile similar to continental Southern European populations (close to Thracians) (Järve et al., 2019; Saag et al., 2025).

f4 tests show that the first Eastern/Central European group forms clades with preceding Illyrian-Thracian populations on the right bank of the Dnieper. qpAdm results indicate that this group can be modeled based on ancestral groups of the Yamnaya culture, the Trypillia culture, and a minor proportion of East Asian populations. The other two groups of Chernyakhov individuals demonstrate a larger proportion of Trypillia genetic heritage (up to 70 %). One representative of the Chernyakhov culture has a pronounced Southern component and clusters in PCA with modern Cypriots (Saag et al., 2025).

Data were also indicate probable connections between bearers of the Chernyakhov culture and early Slavic groups. According to qpAdm modeling data, medieval groups of the Golden Horde period, including presumed Slavs and nomads from the Seversky Donets basin, are formed 100 % from the genetic pool of Group 1 of the Chernyakhov culture, suggesting direct continuity between the Late Antique population of the Chernyakhov culture and the early Slavs (Saag et al., 2025). Data were also obtained indicating genetic continuity through the maternal line between a representative of the Chernyakhov culture from the 3rd century CE (Odessa region) and an individual of Slavic origin from the medieval burial ground “Minino II” from the 12th–13th centuries CE (Vologda region). The individuals were found to have a complete match of the mitochondrial sequence (Rozhdestvenskikh et al., 2025).

Genomic data from individual representatives of Northern Black Sea cultures. The genetic profiles of two individuals from the 8th–9th centuries CE from burials of nomads of unclear cultural attribution at the site “Andreevskaya Shchel” (Anapa) contain, according to qpAdm modeling, a dominant contribution from the population of Armenia of the Middle Bronze Age (76–96 %) (Maróti et al., 2022).

Within the scope of this review, we have systematised archaeogenetic data from individual studies and small samples. Nevertheless, even with limited material, genetic connections are clearly evident both within individual cultures (as in the case of the Sarmatians) and between cultures–among the Alans, Bulgars, and bearers of the Saltovo-Mayaki culture.

**Meta-analysis**

**Fig. 2.**
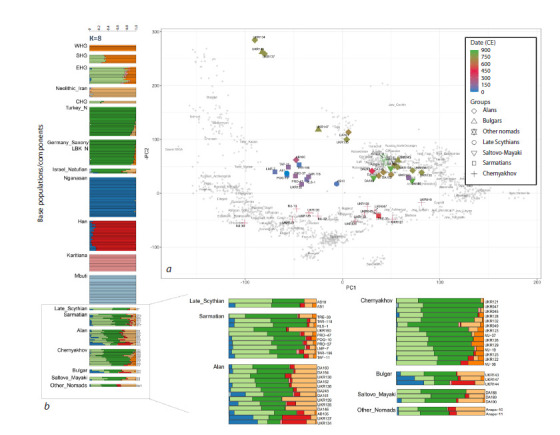
Results of the meta-analysis for individuals from the Northern Black Sea region. a – principal component analysis (PCA); b – population structure analysis (ADMIXTURE) with K = 8 inferred ancestral components. Component (population) designations: WHG – Western Hunter-Gatherer (9,107–5,986 BC); SHG – Scandinavian Hunter-Gatherer (5,967–5,484 BC); EHG – Eastern Hunter-Gatherer (9,649–5,535 BC); Neolithic_Iran – individuals from Neolithic Iran (8,295–7,976 BC); CHG – Caucasus Hunter-Gatherer (11,961–7,599 BC); Turkey_N – individuals from Neolithic Anatolia (9,656–5,835 BC); Germany_Saxony_LBK_N – individuals of the Linear Pottery culture (5,400–4,900 BC); Israel_Natufian – individuals of the Natufian culture (12,000–9,500 BC); Nganasan – present-day Nganasan individuals (Siberia); Han – present-day Han individuals (China); Karitiana – present-day Karitiana individuals (Brazil); Mbuti – present-day Mbuti individuals (Central Africa).

To obtain a comprehensive picture of the population genetic structure of the Northern Black Sea region in the 1st millennium CE, we conducted a meta-analysis of the previously published and above-mentioned whole-genome data using PCA and ADMIXTURE methods. The results of the analysis are presented in Fig. 2.

In the projection of the first two principal components, formed on the basis of modern populations of Western Eurasia and the Caucasus, the earliest samples of the 1st millennium CE (Late Scythian and Sarmatian individuals) occupy an intermediate position between European and Caucasian groups. The Sarmatians form a distinct genetic cluster, consistent with published archaeogenetic data (Saag et al., 2025; Schütz et al., 2025). The only exceptions are two samples: one clusters with the Alans, Bulgars, and bearers of the Saltovo-Mayaki culture, while the other, the latest from this sample (5th–6th centuries CE), clusters with modern Southern European populations.

Representatives of the Chernyakhov culture do not form a single cluster, but are predominantly located near modern Central European and Balkan populations, with one Chernyakhov individual clustering with the Late Scythian and Sarmatian samples (Fig. 2a).

The Alans and Bulgars – representatives of the Saltovo- Mayaki culture – demonstrate pronounced intragroup heterogeneity. A significant portion of these samples cluster within Caucasian populations. Three samples – the Alan from the Ciscaucasus DA161, along with the Alan UKR135 and Bulgar UKR147 from the forest-steppe zone – are located near Central Asian populations. Another three samples – the Alans UKR134, UKR144, and the Bulgar UKR137 from the foreststeppe zone – are notably shifted on the PCA plot towards Eastern populations. It should be noted that the earliest studied Alan from the Ciscaucasus (AB105; 2nd–4th centuries CE) falls within the cluster of Late Scythian and Sarmatian samples, consistent with the hypothesis that the Alans emerged from Sarmatian groups. However, all later representatives of the Alan population show greater similarity to Caucasian populations rather than to steppe Sarmatian groups.

Similar results were obtained from ADMIXTURE analysis (Fig. 2b). The genetic profiles of representatives from each studied culture (Late Scythians/Sarmatians, Alans, Bulgars, and Chernyakhov) exhibit clearly distinguishable differences. In the genetic profiles of the earliest steppe groups (Scythians and Sarmatians), as well as in the early Alans, a small proportion of the Western Hunter-Gatherer component is present which is completely absent in later representatives of the Alans and other bearers of the Saltovo-Mayaki culture. However, this component is present in significant proportions in the Chernyakhov individuals, which testifies to different origins for all these groups inhabiting the Northern Black Sea region during the 1st millennium CE and the absence of direct continuity between them. The genetic profile of the Alans and Bulgars shows contributions from Caucasus Hunter- Gatherers and/ or ancient Iranian Neolithic farmers, indicating a connection of these groups with the ancient population of the Caucasus. Several Bulgar and Alan individuals possess a pronounced Asian component, explaining their shift on the PCA plot towards Eastern populations and suggesting the involvement of migrants from Eastern Eurasia in the formation of these groups and certain representatives of the Northern Black Sea population.

The bearers of the Chernyakhov culture are characterized by significant diversity in genetic profiles, with a common predominance of the Anatolian Neolithic component combined with Western and Eastern Hunter-Gatherer components, which generally reflects their predominantly European genetic profile.

**Fig. 3.**
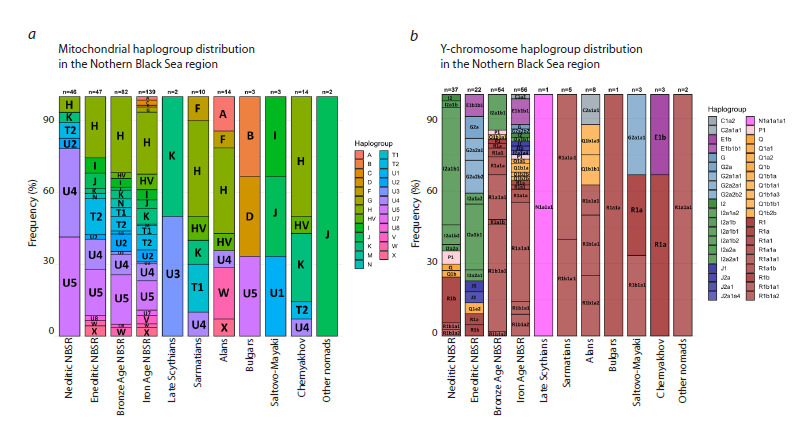
Distribution of mitochondrial (a) and Y-chromosomal haplogroups (b) in the Northern Black Sea region * The figure shows abbreviated haplogroup names for clarity.

Mitochondrial DNA and Y-chromosome analysis. Additionally, to identify potential migration routes of the ancient population of the Northern Black Sea region, we compiled all available data on mitochondrial DNA and Y-chromosomes for the analysis of maternal and paternal lineages. A total of 324 individuals from the Northern Black Sea region across different chronological periods were included in the analyses (Fig. 3, see Supplementary Material)1.

Supplementary Materials are available in the online version of the paper: https://vavilov.elpub.ru/jour/manager/files/Suppl_Aitug_Engl_30_4.pdf


Western Eurasian mitochondrial lineages predominate among the Late Scythians (Juras et al., 2017); among the Sarmatians, these are supplemented by the Eastern haplogroup F, potentially indicating a Central Asian maternal contribution (Comas et al., 2004). There is significant genetic diversity among the Alans and Bulgars, including the presence of East Eurasian mitochondrial haplogroups (Comas et al., 2004; Derenko et al., 2010, 2012): subclades A and F among the Alans and B and D among the Bulgars. This confirms the contribution of Central and East Asian components to their genetic profile. Among representatives of the Chernyakhov culture, haplogroups characteristic of West Eurasian populations dominate (H, K, T2, HV, U4).

It should be noted that, unlike data on autosomal markers and mitochondrial haplogroups, the number of available Y-chromosome data for analysis from individuals of the Northern Black Sea region and adjacent regions remains limited.

From the Bronze Age onwards, a sharp increase in the proportion of haplogroups R1a and R1b has been observed in the Black Sea region, which is thought to correlate with Indo- European expansion and the formation of the main branches of Indo-European languages (Allentoft et al., 2015; Haak et al., 2015). Of particular interest is the Late Scythian sample from Crimea with the Y-chromosome haplogroup N1a1a1a1a2a (N-Z1934) – a lineage absent in earlier complexes of the Northern Black Sea region and widely distributed among contemporary Finno-Ugric populations (Post et al., 2019). This Y-chromosome lineage is currently found only among Russians from the central and Volga-Perm regions (YTree, 2025).

The Alans are characterized by a more heterogeneous Y-chro-mosome composition. Alongside the haplotypes R1b-Z2106, R1b-L23, R1b-M269, and R1b-Z2105, previously identified among Scythian groups of the Early Iron Age in the Northern Black Sea region, they also possess haplotypes Q1b-BZ5214, found in modern Chechens, and C2a-Y11606, found in modern populations of Mongolia and China, as well as in ancient elite groups of the Xiongnu and Avars (YTree, 2025) (see Supplementary Material).

The Bulgars and the people of the Saltovo-Mayaki culture are represented by haplogroup R1a-M417, which had previously been recorded in the Northern Black Sea region. In one individual from the Saltovo-Mayaki culture, a complete match of subclade R1b-M269 was identified with an Alan sample and with several individuals from the Bronze Age of Moldova and the North Caucasus, as well as with Early and Late Scythians. In another individual, lineage G2a-Z6653 was found, matching a Late Antique sample from Georgia (YTree, 2025).

The Chernyakhov culture is represented by haplogroups R1a-Z93, R1a-Z645, and E1b-V13, previously identified among Scythian groups of the Northern Black Sea region, populations of the Late Bronze Age of Moldova, as well as among Thracian and, probably, certain Classical Greek groups (Saag et al., 2025; YTree, 2025).

## Discussion

Despite its key role in trans-Eurasian migration processes during the first millennium CE, the Northern Black Sea region remains, from an archaeogenetic perspective, still poorly and fragmentarily characterized to date. While for certain groups, such as the Sarmatians, the Alans of the mountainous and forest-steppe zones, the Bulgars, and to some extent the population of the Chernyakhov culture, it is possible to provide a preliminary description of their genetic characteristics, for a number of archaeological cultures that inhabited this territory, no data are currently available.

The small sample sizes and limited number of whole-genome samples covering various chronological periods and archaeological cultures significantly impede the reconstruction of demographic processes and reduce the reliability of overarching conclusions. Nevertheless, the systematic review and meta-analysis of the available data allowed us to identify the main features of the genetic structure of the population of the Northern Black Sea region during the 1st millennium CE

In several cases, the transition of archaeological culture was not accompanied by complete population replacement, indicating local genetic continuity. This is clearly demonstrated by data from the site “Solnechnodolsk-4” (Ciscaucasus), where an Alan individual was genetically close to Sarmatians and bearers of the Koban culture from the same site (Andreeva et al., 2025). Similarly, a Late Scythian individual from the Belyaus settlement exhibits a genetic profile similar to the earlier Scythian population of the same region (Andreeva et al., 2025).

At the same time, instances of long-distance migration have been documented: genetic links have been identified between the Sarmatians from the Carpathian Basin and the Southern Urals, providing direct evidence of large-scale migrations across the Northern Black Sea region in the first centuries CE (Schütz et al., 2025).

Our meta-analysis revealed genetic connections between certain representatives of the Sarmatians, Alans, Bulgars, and bearers of the Saltovo-Mayaki culture. The groups from the forest-steppe zone and the Caucasian Alans demonstrate the greatest similarity to each other, which may indicate a common origin or prolonged contacts. At the same time, within the structure of the studied groups, we were able to document the main directions of genetic influences: Caucasian and East Eurasian, which is consistent with our results from the analysis of uniparental markers (mitochondrial DNA and Y-chromosome).

The genetic diversity identified among individuals of the Chernyakhov culture is complemented by data from the sites “Shishaki” and “Minino II”, where genetic connections with later Slavic groups are observed (Rozhdestvenskikh et al., 2025; Saag et al., 2025). These results provide a foundation for further, more in-depth genetic analysis of the processes underlying the formation of Slavic groups in Eastern Europe.Finally, it was demonstrated that the Sarmatians who arrived in the Black Sea region were not genetically close to the local Scythians (Andreeva et al., 2025). This suggests that the Scythian population was largely replaced by the Sarmatians without genetic admixture.

It should be noted that, as a result of our analysis using previously published genomic data, we did not identify traces of admixture between the various ethnic and cultural groups inhabiting the Northern Black Sea region during the 1st millennium CE

## Conclusion

The systematic review and meta-analysis of archaeogenetic data on the population of the Northern Black Sea region during the 1st millennium CE allow us to view the region not as a sequence of successive cultures, but rather as a long-functioning contact zone. The obtained data have created a solid foundation for subsequent expansion of sample sets and deepening of research. A promising direction is the acquisition of wholegenome data for the key populations of the Northern Black Sea region for which genomic data are still absent, including the population of the Bosporan Kingdom, the nomadic confederations of the early Middle Ages (Goths, Huns, etc.), early Slavic groups, as well as the populations of Bulgaria and the Khazar Khaganate.

Investigating the connections between the genetic characteristics of the population of this region, the transmission of cultural traditions, and the spread of languages is particularly important for clarifying its demographic history in subsequent historical periods (the Middle Ages). This requires a comprehensive interdisciplinary approach involving geneticists, archaeologists, anthropologists, and linguists.

## Conflict of interest

The authors declare no conflict of interest.
